# Variation in Intrinsic Aerobic Capacity Reveals Distinct Differences in the Maintenance of Rat Knee Joint Integrity

**DOI:** 10.1002/jor.70299

**Published:** 2026-10-19

**Authors:** Darsh Tripathi, Mauricio Delgado‐Bravo, Ruth A. Seerattan, Venus Joumaa, David A. Hart, Raylene A. Reimer, Lauren Gerard Koch, Walter Herzog

**Affiliations:** ^1^ Human Performance Laboratory, Faculty of Kinesiology University of Calgary Calgary Alberta Canada; ^2^ McCaig Institute for Bone and Joint Health University of Calgary Calgary Alberta Canada; ^3^ Carrera de Kinesiología, Departamento de Ciencias de la Salud Facultad de Medicina, Pontificia Universidad Católica de Chile Santiago Chile; ^4^ Department of Surgery University of Calgary Calgary Alberta Canada; ^5^ Department of Biochemistry and Molecular Biology, Cumming School of Medicine University of Calgary Calgary Alberta Canada; ^6^ Department of Physiology and Pharmacology College of Medicine and Life Sciences, The University of Toledo Toledo Ohio USA

**Keywords:** high‐fat high‐sucrose diet, infrapatellar fat pad, intrinsic aerobic capacity, knee joint integrity, metabolic syndrome, obesity, osteoarthritis, subchondral bone, synovial membrane

## Abstract

The objective was to test how intrinsic aerobic capacity affects the articular cartilage, infrapatellar fat pad (IFP), subchondral bone, and synovial membrane of the rat knee joint. Thirty‐five 24‐week‐old male N:NIH rats selectively bred for high aerobic capacity (high‐capacity‐runner [HCR]; *n* = 17) and low aerobic capacity (low‐capacity‐runner [LCR]; *n* = 18) were fed either a high‐fat/high‐sucrose (HFHS) diet or a chow diet for 12 weeks. Alterations in articular cartilage, subchondral bone microarchitecture, IFP hypertrophy, and synovial membrane cytokine levels were quantified. Body composition, serum cytokines, and glucose tolerance were assessed at Week 12. Compared to HCR rats, LCR rats had higher modified Mankin scores, bone volume, total volume, bone mineral density, bone volumetric fraction, and trabecular thickness, and lower specific bone surface, bone surface density, and connectivity density. LCR rats also had higher RANTES (Regulated on Activation, Normal T Cell Expressed and Secreted) and lower EGF (epidermal growth factor) levels in synovium analytes. Furthermore, in response to the HFHS diet, there were no differences in adipocyte hypertrophy within the IFP for either the HCR or LCR rats. LCR rats demonstrated hyperglycemia, elevated pro‐inflammatory marker levels, and decreased anti‐inflammatory marker levels compared to HCR rats. It was concluded that intrinsic high aerobic capacity maintains superior knee joint integrity both with and without an obesogenic dietary challenge, likely due to superior metabolic resistance. Whether intrinsic high aerobic capacity might protect joint degeneration in obesity and metabolic syndrome in other species including humans, warrants investigation.

## 1 Introduction

There has been limited research regarding the interplay between obesity, metabolic syndrome, and osteoarthritis (OA) [1]. Often, the incidence of knee joint OA in patients with obesity has been attributed to mechanical factors, such as increased joint loading resulting from excess body weight [2, 3]. However, the high prevalence of OA in non‐weight‐bearing joints of the hand and wrist in individuals with obesity suggests that factors other than mechanical stress, such as local and systemic inflammation, and metabolic syndrome, may play a role in the development and progression of knee joint OA [1, 4, 5, 6, 7, 8, 9, 10]. The high incidence of OA in patients with obesity has led to the definition of a distinct subtype of OA, labeled metabolic OA [10, 11].

To investigate metabolic OA, our lab developed a pre‐clinical model of diet‐induced obesity where Sprague Dawley (SD) rats fed a high‐fat, high‐sucrose (HFHS) diet became obese and developed metabolic syndrome resulting in the reliable onset of knee degeneration [12, 13, 14]. Since then, there has been a series of studies on dietary and exercise interventions aimed at slowing or preventing metabolic disturbances and joint degeneration in animals exposed to HFHS diets [13, 14, 15, 16, 17]. These interventions produced two remarkable findings. First, aerobic exercise interventions prevented the development of knee joint OA in HFHS‐fed SD rats, even though they gained body weight and exhibited body fat percentages similar to their sedentary counterparts on the HFHS diet [18, 19]. Second, muscles with high aerobic capacity, such as the soleus, were protected from degenerative changes in the presence of an HFHS diet, while muscles with low aerobic capacity, such as the vastus lateralis, showed substantial fibrosis, fat infiltration, and local inflammation [20, 21]. These results suggested that tissues and animals with a high aerobic capacity might be protected from obesity‐ and metabolic syndrome‐associated musculoskeletal degeneration, including the development of knee joint OA.

To investigate the effect of intrinsic aerobic capacity in a preclinical model, N:NIH rats were selectively bred for high (HCR) and low (LCR) aerobic capacity over 49 generations based solely on their untrained treadmill running capacity [22, 23, 24, 25]. This selective breeding process has concentrated genes responsible for divergent aerobic capacity, creating rat lines that differ in intrinsic metabolic phenotype without any exercise training. Importantly, studies have shown that LCR rats exhibit multiple metabolic syndrome factors even when maintained on standard chow diets from a young age, including higher body weight, elevated fasting glucose and insulin, impaired glucose tolerance, higher visceral adiposity, hepatic steatosis, reduced mitochondrial content and oxidative capacity in skeletal muscle and liver, and shortened lifespan compared to HCR rats [22, 23, 24, 25]. In contrast, HCR rats display a metabolically healthy phenotype on a chow diet, with no cardiovascular or metabolic syndrome risk factors, enhanced mitochondrial function, greater energy expenditure, and superior metabolic resilience [22, 23, 24, 25, 26, 27, 28].

The purpose of this study was to assess whether differences in intrinsic aerobic capacity lead to changes in the knee joint (cartilage, infrapatellar fat pad [IFP], synovial membrane, subchondral bone) in an established pre‐clinical rat model of diet‐induced obesity. It was hypothesized that HCR rats will have greater knee joint integrity compared to LCR rats, and that the metabolic differences between HCR and LCR rats, irrespective of diet, explain the differential susceptibility to knee joint degeneration in these rat populations.

## 2 Methods

### 2.1 Animals and Intervention Protocol

Thirty‐five male, 24‐week‐old N:NIH rats (LCR; *n* = 18, and HCR; *n* = 17; Exercise Rat Resources, The University of Toledo, Toledo, Ohio, United States) were housed in pairs on a 12 h dark/light cycle. The animals of each phenotype were randomized to either a HFHS diet (20% of total weight as fat, 50% sucrose, 20% protein, and 10% fiber and micronutrients; custom diet #102412, Dyets Inc., Bethlehem, Pennsylvania, United States) or a chow control diet (5% of total weight as fat, 47.5% carbohydrates (only 4% from sucrose), 25% protein fiber and micronutrients, and 10% moisture; Lab Diet 5001, United States) for 12 weeks. All experiments were approved by the University of Calgary Life and Environmental Sciences Animal Care Committee (#AC20‐0069), and all studies were conducted in accordance with the animal welfare regulations and guidelines at the University of Calgary.

### 2.2 Preparation of Knee Joints

At the end of the 12‐week diet intervention period, rats were anesthetized using 5% isoflurane, then euthanized by severing the aorta and vena cava. Knee joints were harvested, excess muscles were removed, and the left knee joints were fixed in a 10% neutral buffered formalin solution for 4 weeks at room temperature. The knee joints were scanned using a micro‐computed tomography system prior to decalcification in Cal‐x‐II (Fisher Scientific) at room temperature for 4 weeks. Knee joints were dehydrated in alcohol, cleared in xylene, and infiltrated with paraffin using a Leica TP1020 automated processor (Leica Biosystems, Nussloch, Germany). Knee joints were then embedded in a mix of Paraffin wax (Fisher Scientific). Serial sagittal sections of 10 μm thickness were obtained and mounted. Alternate slides were stained with Hematoxylin, Fast Green, and Safranin‐O.

### 2.3 Knee Joint Mankin Scoring

Sections were scored by two independent, blinded observers, with a high degree of concordance across all slides; when scores differed by more than one point, the slides were jointly re‐reviewed to reach consensus, and the final score for each slide was defined as the average of the two evaluators' ratings. A modified‐Mankin Score was used, where five criteria were assessed on the standard 14‐point Mankin scale: cartilage integrity 0−6; cellular changes 0−3; loss of proteoglycan 0−3; and tidemark integrity 0−2 [29]. The total score for each knee was obtained by summing the scores from the patella, femoral groove, and the tibia and femur in both the medial and lateral compartments [29]. Scores for synovium thickening (0−4) and meniscus damage (0−3) were then added [29].

### 2.4 IFP Analysis

Four unstained slides, with serial sagittal sections of the whole knee joint (above) were selected from the midline of the joint and used for this analysis. Sections, at least 200 μm apart, which showed a fully intact sagittal view of the IFP and contained at least 2000 adipocytes, were stained with Picro Sirius Red to determine collagen content and fat cell size distribution. Images were captured with a ×10 objective on an Olympus BX53 microscope, using an Olympus DP73 camera and the Olympus Cell‐Sens Standard imaging system. Adipocyte size (*n* = 2000 cells) was determined using ImageJ software (Rasband, W.S., ImageJ, U.S. National Institutes of Health, Bethesda, Maryland, USA).

### 2.5 Synovial Membrane Preparation

The synovial membrane was pulverized using a mortar and pestle pre‐cooled in liquid nitrogen, and solubilized in ice cold‐protein extraction buffer (50 mM Tris, 150 mM NaCl, 20 mM Na4P2O7, 5 mM EDTA, 1 mM DTT, 0.1% Triton X‐100, and 1 tablet of protease inhibitors [Complete, Roche Diagnostics, Quebec, Canada] per 50 mL of solution, pH = 7.4) at a ratio of 5 µL per 1 mg of synovial membrane powder. Samples were then sonicated for 30 min at 4°C and centrifuged at 13,000 rpm for 15 min. The protein concentration in the supernatant was assessed using a bicinchoninic acid assay (BCA) kit (Pierce BCA Protein Assay Kit, Thermo Fisher Scientific, Illinois, USA). The synovial membrane protein extracts were aliquoted in 50 µL volumes at a concentration of 2 mg/mL, and analyzed for their adipokines and cytokines using a 27 Multiplex Discovery Assay with LuminexxMAP technology (Eotaxin, EGF, Fractalkine, IL‐1α, IL‐1β, IL‐2, IL‐4, IL‐5, IL‐6, IL‐10, IL‐12(p70), IL‐13, IL‐17A, IL‐18, IP‐10/CXCL10, GRO/KC, IFN‐γ, TNF‐α, G‐CSF, GM‐CSF, MCP‐1, leptin, LIX, MIP‐1α, MIP‐2, RANTES, VEGF; Eve Technologies, Calgary, Canada).

### 2.6 Micro‐Computed Tomography

The micro‐CT system used in this study was a cone‐beam tomograph (μCT 35, Scanco Medical, Brüttisellen, Switzerland). Specimens were scanned in the transverse plane mounted in a cylindric sample holder at an isotropic resolution of 30 μm^3^ voxel size (144 μA; 55 keV). The volume of interest was determined manually for the distal femur epiphysis and the proximal tibial epiphysis and included the entire subchondral bone region. A Gaussian filtering (sigma of 0.8, support of 1) was used to smooth the data and reduce noise. Total volume (TV), bone volume (BV), bone surface (BS), bone volume fraction (BVF), specific bone surface (SBS), trabecular number (Tb.N), trabecular thickness (Tb.Th), trabecular separation (Tb.Sp), bone mineral density (BMD), tissue mineral density (TMD), bone surface density (BSD), and connectivity density (Conn.D) were quantified according to established guidelines for rodent bone microstructure assessment [30].

### 2.7 Body Composition

Body composition was assessed as described previously [31]. Briefly, during the 12‐week intervention period, rats were lightly anaesthetized with isoflurane, and body composition was measured using Dual X‐ray absorptiometry (DXA) with specialized small rodent software (GE Lunar). DXA scans were performed at 0, 3, 6, 9, and 12 weeks of the diet intervention. Body mass was measured at the beginning of each week.

### 2.8 Oral Gavage Glucose Tolerance Test

An oral glucose tolerance test (OGTT) was conducted following overnight food deprivation at the end of the diet intervention at 12 weeks. Blood was collected via tail nick at 0, 15, 30, 60, and 120 min post‐oral gavage of 2 g/kg glucose. Blood glucose was measured immediately with a blood glucose meter (OneTouch Verio and Blood Glucose Monitoring System, Lifescan, Switzerland).

### 2.9 Blood Cytokines and Adipokines

One week prior to the start of the 12‐week intervention protocol and following 16 h of food deprivation, blood was collected via tail nick to evaluate the baseline values for markers of metabolic syndrome. Blood samples were also collected after 12 weeks of the diet intervention. Serum samples were stored at −80°C. Serum cytokines were quantified using a 22‐plex Rat Multiplex Discovery Assay with LuminexxMAP technology (Eotaxin, EGF, Fractalkine, IL‐1β, IL‐2, IL‐4, IL‐5, IL‐6, IL‐12(p70), IL‐13, IL‐17A, IP‐10, IFN‐γ, TNF‐α, G‐CSF, GM‐CSF, MCP‐1, LIX, MIP‐1α, MIP‐2, RANTES, VEGF; Eve Technologies, Calgary, AB, Canada).

### 2.10 Statistical Analysis

The sample size was calculated a priori based on the results of Mankin scores from a previous study, using the software GPower 3.1.9.7 [32, 33]. A two‐way ANOVA was used with main effects of diet (HFHS and chow) and strain (HCR and LCR) and their interaction. If a significant interaction effect was found, a one‐way ANOVA with Bonferroni post hoc test was performed to determine differences between groups. Since diet did not affect modified‐Mankin scores, diet groups were collapsed within each strain for synovial membrane cytokine analysis. A two‐tailed *t*‐test was used to compare synovial membrane cytokine levels across the two strains. A two‐way repeated measures ANOVA with Bonferroni correction was used to assess blood glucose across time in the experimental groups. Analysis was conducted using IBM SPSS Statistics V26 (IBM SPSS, Armonk, NY). Significance was accepted for *p* < 0.05, two‐tailed test.

## 3 Results

### 3.1 Knee Joint Integrity

#### 3.1.1 Modified‐Mankin Scores

There was a significant main effect of strain on knee joint integrity with HCR rats having significantly lower modified‐Mankin scores than did the LCR rats (Figure 1; F_1,31_ = 13.15, *p* = 0.001). There was no significant diet effect or interaction effect.

**Figure 1:**
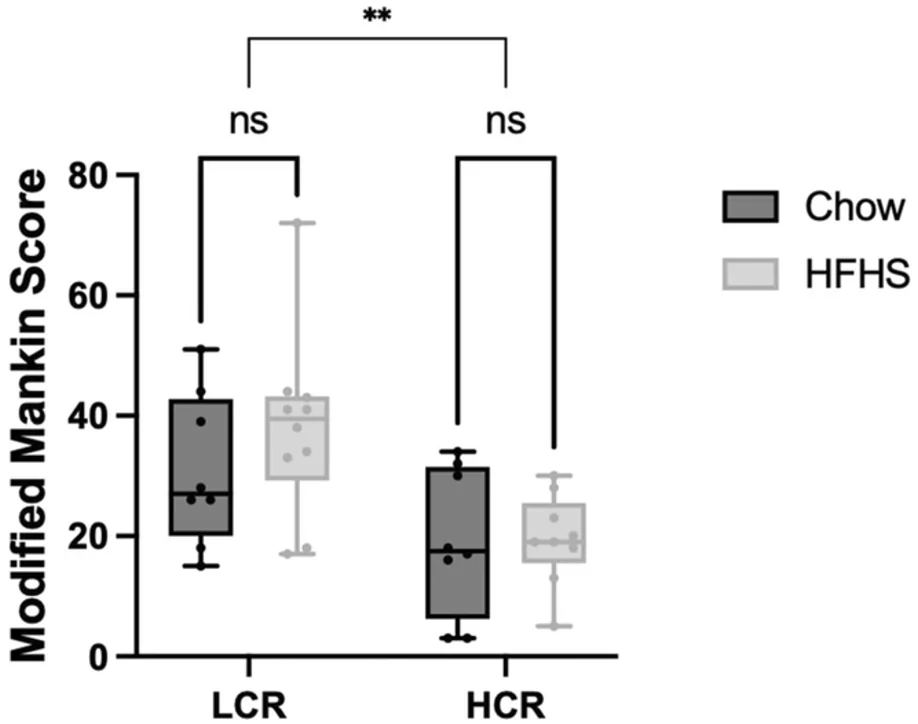
Modified Mankin score: The modified Mankin score was obtained as the algebraic sum of the 14‐point “classic” Mankin scores from six knee joint sites (medial tibial plateau, medial femoral condyle, lateral tibial plateau, lateral femoral condyle, patella, and femoral groove) and the scores for the synovium and meniscus. The LCR rats fed the chow diet had mean modified Mankin scores of 30.9 (95% CI: 20.3−41.4), and the LCR rats fed the HFHS diet had mean modified Mankin scores of 38.1 (95% CI: 27.1−49.1). The HCR rats fed the chow diet had mean modified Mankin scores of 19.1 (95% CI: 8.9−29.3), and the HCR rats fed the HFHS diet had mean modified Mankin scores of 19.4 (95% CI: 13.7−25.2). **p < 0.005 between strains.

#### 3.1.2 Histology

Cavitation/cysts were observed in the articular cartilage across all groups (Figure 2). The cysts are indicative of cavitation which is defined as the formation of a cyst or fluid‐filled space within cartilage [34]. There was no significant main effect (Figure 2; F_1,31_ = 0.15, *p* > 0.05), no significant diet effect (Figure 2; F_1,31_ = 0.93, *p* > 0.05), and no significant interaction effect (Figure 2; F_1,31_ = 0.85, *p* > 0.05) in the number of cysts across all groups. Because of the sample preparation methods used, it was not possible to identify the original content of the cysts. Future work is aimed at identifying the content and more detailed pathology of these cysts.

**Figure 2:**
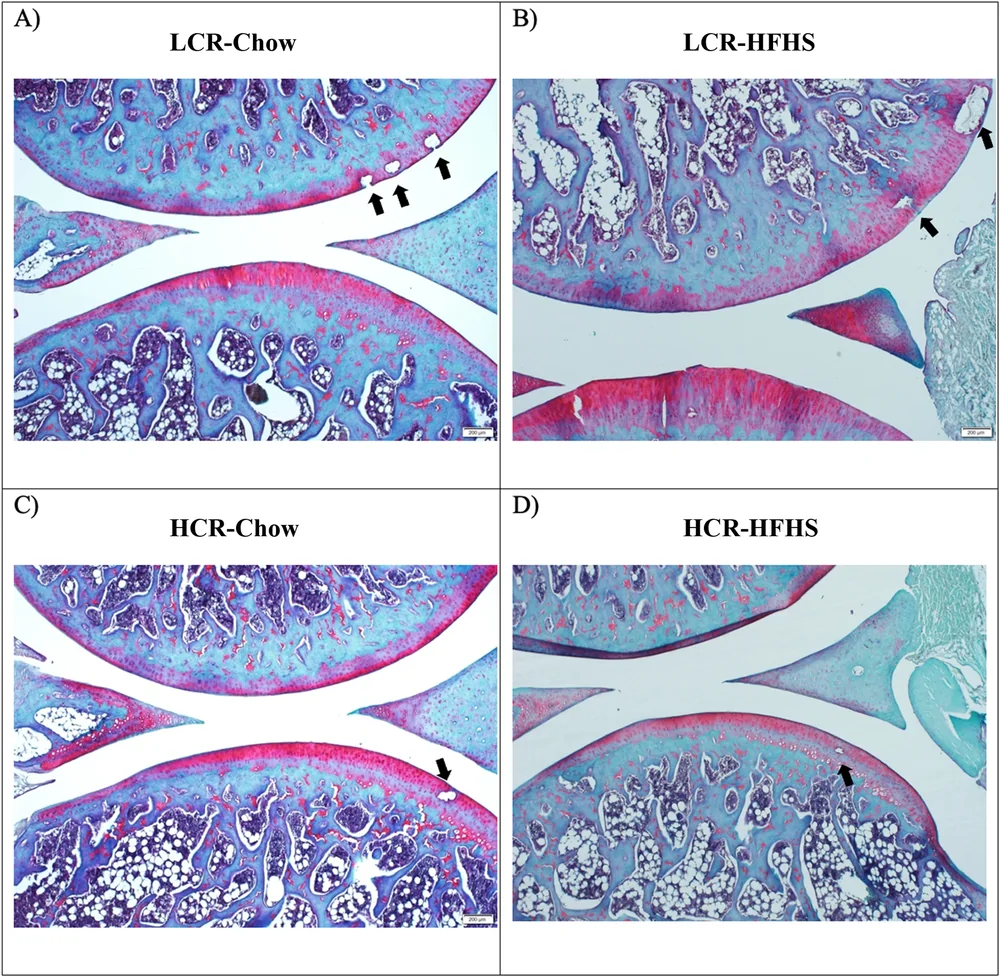
Sagittal plane histological sections of the tibio‐femoral joint with femur on top and tibia at the bottom. (A) LCR rats fed a chow diet. (B) LCR rats fed a HFHS diet. (C) HCR rats fed a chow diet. (D) HCR rats fed a HFHS diet. The black arrows point to articular cartilage cysts.

#### 3.1.3 Cell Hypertrophy Analysis in the IFP

The IFP in the LCR and HCR rats, irrespective of diet, showed no statistical differences in adipocyte size between the groups.

#### 3.1.4 Synovial Membrane Cytokine Levels

Synovial membrane analytes did not differ between chow‐ and HFHS‐fed animals within either strain. However, when LCR and HCR animals were compared regardless of diet, EGF was consistently lower (*p* = 0.02, Figure 3) and RANTES consistently higher (*p* = 0.005, Figure 3) in LCR animals than in HCR animals. There were no other significant differences in the levels of the remaining cytokines assessed between the groups.

**Figure 3:**
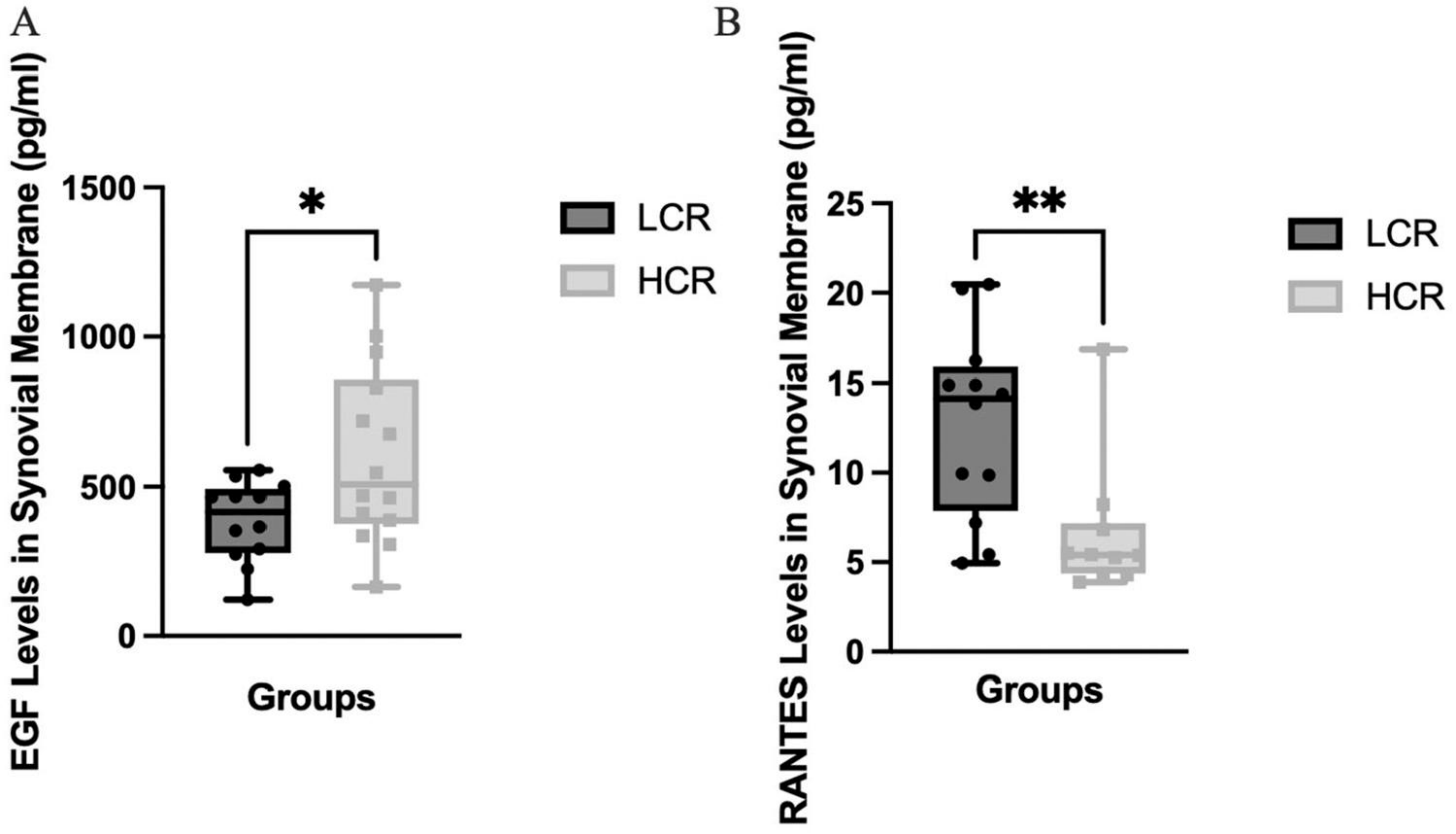
(A) Epidermal growth factor (EGF) levels in the synovial membrane. The LCR rats had mean EGF levels of 385 pg/mL (95% CI: 299−471 pg/mL) compared to the HCR rats which had mean EGF levels of 603 pg/mL (95% CI: 431−774 pg/mL). (B) RANTES levels in the synovial membrane. The LCR rats had mean RANTES levels of 12.7 pg/mL (95% CI: 9.4−16.0 pg/mL) compared to HCR rats which had mean RANTES levels of 6.6 pg/mL (95% CI: 3.9−9.3 pg/mL). *p < 0.05, **p < 0.005.

#### 3.1.5 Subchondral Bone Parameters

The LCR rats, irrespective of diet, had significantly higher BV, TV, BMD, BVF, and Tb.Th, and lower SBS, BSD, and Conn.D, compared to HCR rats (Figure 4, Supporting Information S1: Table 1).

**Figure 4:**
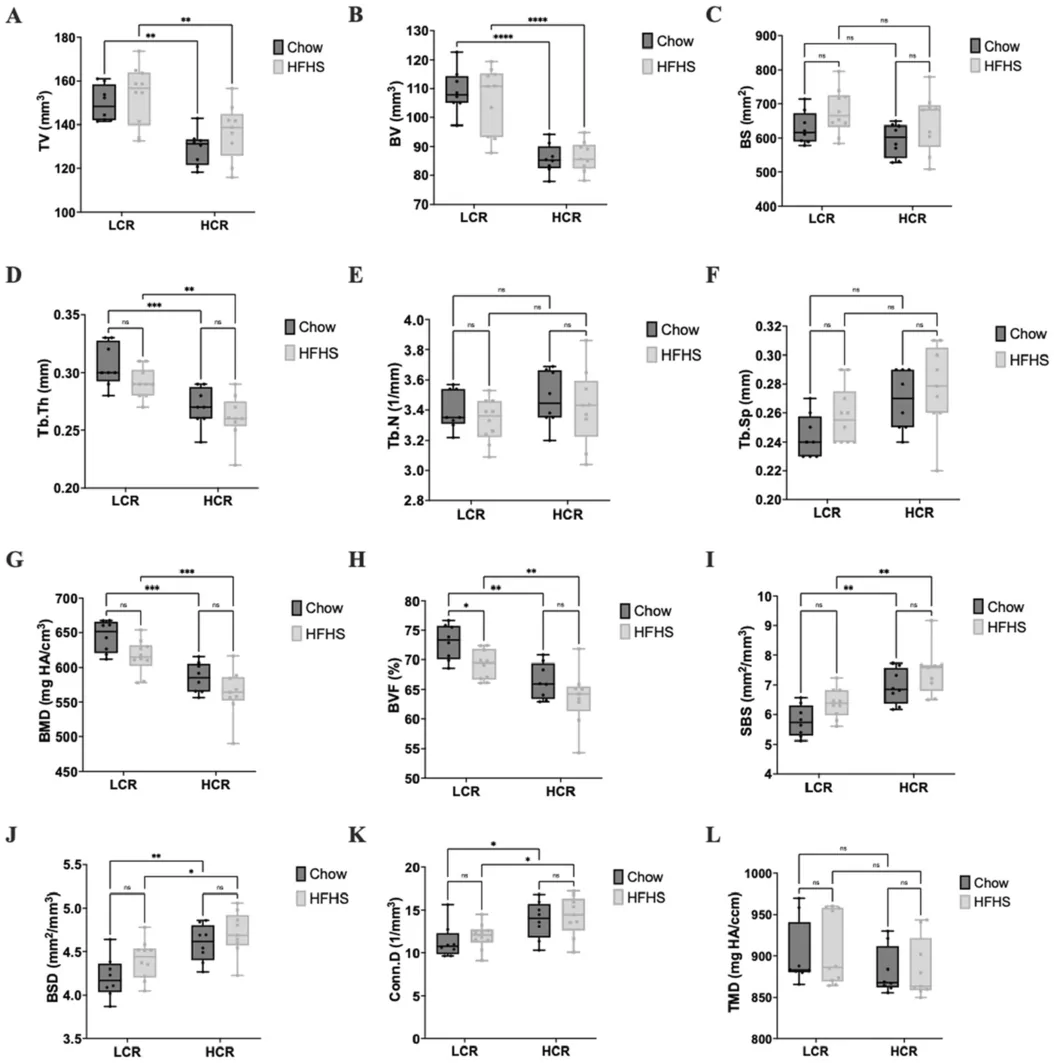
Subchondral bone parameters obtained using MicroCT for all experimental groups at the end of the 12‐week diet intervention. Parameters include: (A) total volume (TV), (B) bone volume (BV), (C) bone surface (BS), (D) trabecular thickness (Tb.Th), (E) trabecular number (Tb.N), (F) trabecular separation (Tb.Sp), (G) bone mineral density (BMD), (H) bone volume fraction (BVF), (I) specific bone surface (SBS), (J) bone surface density (BSD), (K) connectivity density (ConnD.), and (L) tissue mineral density (TMD). *p < 0.05, **p < 0.01, ***p < 0.005, ****p < 0.0001, ^ns^ represents not significant.

### 3.2 Metabolic Disturbances

#### 3.2.1 Body Composition

LCR group rats weighed more than HCR group rats, independent of diet throughout the diet intervention (Figure 5; *p* < 0.0001). Following the 12‐week diet intervention, the LCR and HCR group rats fed the HFHS diet had a significantly greater percentage of body fat (44 ± 5% and 41 ± 6%, respectively) than the corresponding rats on the chow diet (30 ± 6% and 29 ± 4%, respectively; Figure 5).

**Figure 5:**
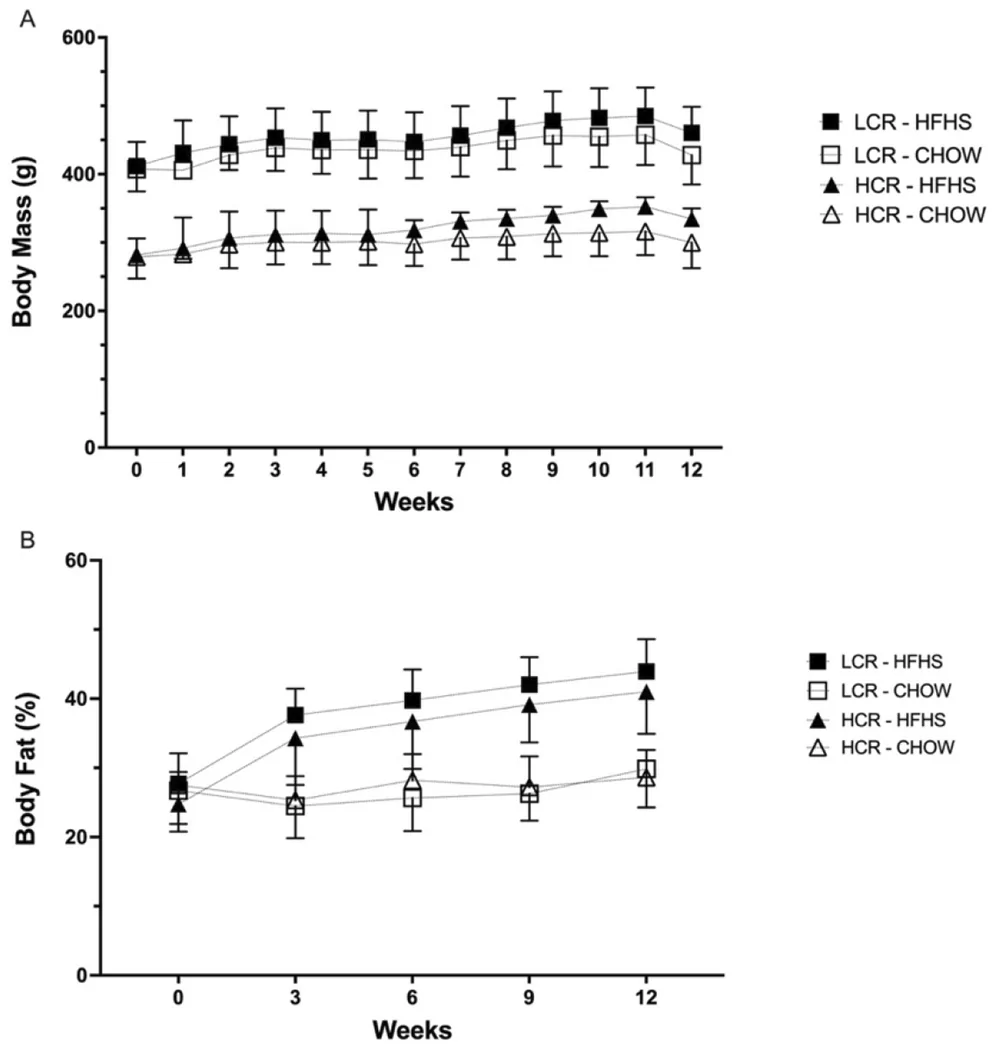
Body mass and body fat percentage for all experimental groups across the 12‐week diet intervention. Of note, independent of diet exposure, the LCR group rats had greater body mass than the HCR rats (A), and the LCR and HCR rats increased their body fat percentage to a similar degree when exposed to the HFHS diet (B).

#### 3.2.2 Glucose Recovery

There was a significant main effect of diet (Figure 6A; F_1,30_ = 14.79, *p* < 0.001), with an increase in glucose in the HFHS compared to the chow diet group rats. There was no significant main effect of strain and no interaction effect.

**Figure 6:**
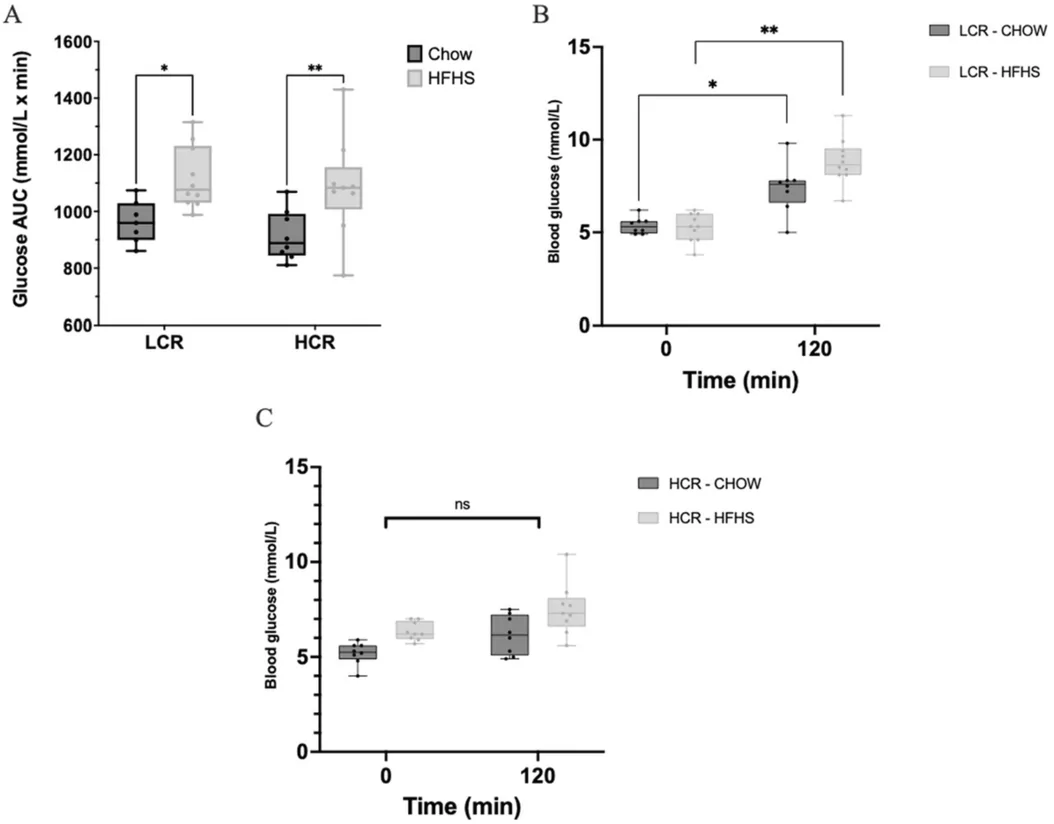
(A) Glucose area under the curve (AUC) values in LCR and HCR rats during the OGTT conducted after the 12‐week diet intervention. The glucose AUC represents the glucose in the bloodstream after administration of the oral glucose load. LCR rats fed the HFHS diet had mean glucose area under the curve (AUC) value of 1118 mmol/L × min (95% CI: 1040−1197 mmol/L × min) which was significantly higher (*p* = 0.015) than LCR rats fed the chow diet which had mean glucose AUC value of 963 mmol/L × min (95% CI: 894−1031 mmol/L × min). HCR rats fed the HFHS diet had a mean glucose AUC value of 1086 mmol/L × min (95% CI: 949−1222 mmol/L × min) which was significantly higher (*p* = 0.008) than HCR rats fed the chow diet which had a mean glucose AUC value of 916 mmol/L × min (95% CI: 841−990 mmol/L × min). (B) LCR rats fed a chow diet had mean blood glucose values of 5.36 mmol/L (95% CI: 4.99−5.73 mmol/L) at baseline compared to 7.40 mmol/L (95% CI: 6.26−8.54 mmol/L) at 120 min of the OGTT. LCR rats fed the HFHS diet had mean blood glucose values of 5.26 mmol/L (95% CI: 4.72−5.80 mmol/L) at baseline compared to 8.83 mmol/L (95% CI: 7.95−9.71 mmol/L) at 120 min of the OGTT. (C) HCR rats fed the chow diet had mean blood glucose values of 5.19 mmol/L (95% CI: 4.70−5.68 mmol/L) at baseline compared to 6.16 mmol/L (95% CI: 5.30−7.03 mmol/L) at 120 min of the OGTT. HCR rats fed the HFHS diet had mean blood glucose values of 6.34 mmol/L (95% CI: 5.98−6.71 mmol/L) at baseline compared to 7.51 mmol/L (95% CI: 6.46−8.56 mmol/L) at 120 min of the OGTT. *p < 0.05, **p < 0.005.

Blood glucose levels remained elevated at 120 min compared to baseline in LCR rats fed the chow or the HFHS diet (Figure 6B, *p* = 0.031 and *p* = 001 respectively). Blood glucose levels at 120 min following the glucose load did not differ from baseline levels in the HCR rats on the chow or the HFHS diet (Figure 6C, *p* = 0.123 and *p* = 0.574, respectively).

#### 3.2.3 Serum Cytokine Levels

No statistical differences were detected for the assessed cytokines between the groups at baseline. At the end of the diet intervention, significant strain, diet, and interaction effects were observed (Figure 7, Supporting Information S1: Table 2). Irrespective of diet LCR rats had significantly higher IL‐1β (interleukin‐1β) and VEGF serum levels (*p* = 0.02 and *p* = 0.03, respectively; Figure 7, Supporting Information S1: Table 2). LCR rats fed a HFHS diet exhibited significantly lower serum EGF levels (*p* = 0.009; Figure 7, Supporting Information S1: Table 2).

**Figure 7:**
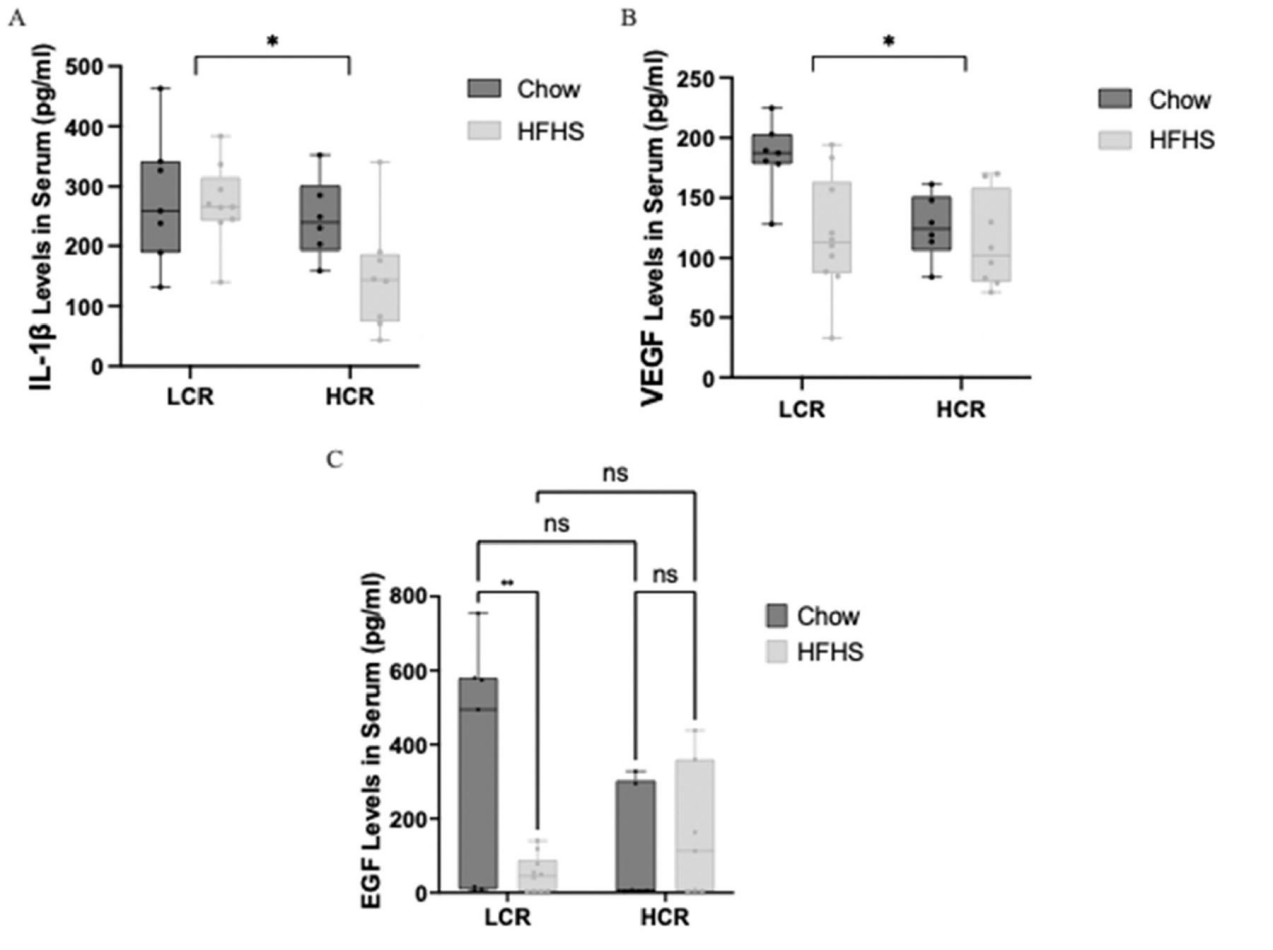
Summary of serum cytokine, IL‐1β (A), and growth factor protein, VEGF and EGF (B, C) levels. *p < 0.05, **p < 0.005.

## 4 Discussion

The primary finding of this study was that intrinsic aerobic capacity was associated with distinct differences in the knee joint cartilage, synovial membrane, and subchondral bone in age‐matched HCR and LCR rats, independent of diet (Figures 1, 3, and 4). There was no detectable induction of hypertrophy of cells in the IFP at baseline or following the HFHS diet exposure in these animals. These differences in knee joint integrity were associated with differences in body mass, serum cytokines, and glucose tolerance, parameters that, in addition to the crucial difference in intrinsic aerobic capacity between the two strains, may have been among the key drivers behind these biological differences (Figures 5, 6, 7).

HCR rats had significantly lower modified‐Mankin scores compared to LCR rats, indicating better retention of knee joint integrity, irrespective of diet. The similar body‐fat percentages in the HCR and LCR rats fed the HFHS diet suggest that total adiposity per se is not sufficient to explain the difference in the modified‐Mankin scores between the HCR and LCR group rats. Instead, the data suggest that the divergent metabolic phenotype of the HCR rats, of which high intrinsic aerobic capacity is a well‐defined central feature, may affect the host response to excess caloric intake such that they can store fat without transitioning into the metabolically unhealthy, pro‐inflammatory phenotype, as seen in LCR rats.

This result might be compared to a previous study in SD rats where aerobic training over a 12‐week period also did not prevent excess body fat accumulation when the rats were exposed to an HFHS diet, but alleviated metabolic disturbances and prevented the knee joint degeneration observed in the HFHS group cohort that was not exposed to the aerobic exercise [18]. Such observations led to the nomenclature of metabolically healthy obese animals compared to metabolically compromised obese animals. However, the present study differed from the previous studies with SD rats in that, in the present study, the intrinsic high aerobic capacity was sufficient to maintain knee joint integrity, while the protection in the previous studies was provided through aerobic training.

Hence, the lower knee joint integrity in the LCR rats than the HCR group rats, quantified by the elevated modified‐Mankin scores, may be associated with other local and systemic differences. To pursue this concept of involvement of other local agents, adipocyte hypertrophy in the IFP and synovial membrane cytokine levels were assessed. There were no significant differences in adipocyte hypertrophy detected between the HCR and LCR rats. This is in contrast to findings in SD rats, where significant hypertrophy in the IFP was noted after 12 weeks on the same HFHS diet as used in this study [35, 36]. Alterations in the IFP have been linked to the development and the rate of progression of knee OA in humans [37]. Since there was no activation of adipocytes in the IFP in the HCR or LCR rats, the increased Mankin scores for the LCR rats compared to the HCR rats may have a different origin than the IFP.

The cytokine profile within the synovial membrane showed increased levels of EGF in HCR rats and increased levels of RANTES in the LCR rats, suggesting that the divergent phenotype distinguishing these strains, of which intrinsic aerobic capacity is a central feature, is accompanied by differences in the joint inflammatory response. EGF is known to act on synoviocytes and chondrocytes via EGF receptor signaling leading to increased cell proliferation, cell survival, and stem cell maintenance [38]. In addition, another study has reported that EGF receptor‐deficient mice developed more severe OA than control mice, as indicated by fewer superficial chondrocytes, less secretion of boundary lubrication, and weaker mechanical strength of the cartilage surface [39]. The decreased levels of EGF in the synovial membranes of the LCR rats, thus, may reflect a reduced ability to maintain joint homeostasis, exposing them to risk for greater knee joint deterioration. Serum EGF levels did not differ between strains, though a reduction was observed specifically in LCR rats fed a HFHS diet, potentially reflecting impaired systemic ability to respond to obesity‐induced inflammation. In contrast, RANTES, a CC‐chemokine critically involved in the recruitment of monocytes, T‐lymphocytes, and natural killer cells via CCR1, CCR3, and CCR5 receptors, was elevated in the synovial membrane of LCR rats, pointing to a chemokine‐driven leukocyte infiltration pattern consistent with an inflammatory environment in the knee joint [40]. Notably, serum RANTES levels remained comparable across groups, despite their synovial level increases in LCR rats. In contrast, SD rats exposed to the HFHS diet had elevated systemic RANTES levels, highlighting that the absence of aerobic capacity stratification in other models may affect joint‐specific inflammatory responses that are only apparent when intrinsic aerobic capacity is a controlled variable [41].

The involvement of subchondral bone in the pathophysiology of knee OA remains to be fully understood, and therefore, research surrounding this tissue is critical [42]. The cellular crosstalk between cartilage and subchondral bone is thought to be a key modulator of joint homeostasis, and changes in this crosstalk have been thought to be detrimental to system integrity [43, 44]. In the present study, distinct structural differences in subchondral bone microarchitecture between HCR and LCR rats were detected, reflecting inherent phenotypic differences between the two strains. These findings suggest that subchondral bone characteristics may depend, in part, on intrinsic aerobic capacity. If these different subchondral bone characteristics are directly or indirectly associated with aerobic capacity, elucidating the mechanistic pathways that are potentially affected by aerobic capacity requires further investigation. Notably, the bone remodeling response in the LCR rats is similar to what has been reported for human OA knee joints [45, 46, 47], and when considered alongside their higher modified Mankin scores and signs of synovial metabolic dysregulation, this pattern in the LCR rats may reflect early degenerative remodeling rather than an adaptive response.

Studies have shown that LCR animals are metabolically vulnerable even on a chow control diet, and that the dietary challenge of an HFHS diet results in further metabolic disturbances [27]. The present study demonstrated that LCR rats fed the HFHS diet gained significantly more body fat compared to LCR rats fed the chow diet. However, other metabolic parameters such as serum cytokine levels and glucose regulation were comparable between the LCR rats, irrespective of diet.

At baseline, serum cytokine levels were comparable between LCR and HCR rats. After the 12‐week diet HFHS intervention, the LCR rats, independent of diet, exhibited higher serum levels of IL‐1β and VEGF, compared to the HCR rats. These elevated cytokine levels may be indicative of a low‐level systemic inflammatory state in the LCR rats compared to the HCR rats. In contrast, in SD rats, the HFHS diet appeared to lead to an overt systemic low‐inflammatory state [16, 35, 48]. In a joint, IL‐1β has been shown to contribute to cartilage degradation by inducing matrix metalloproteinases that can degrade collagen type II and proteoglycans, molecules that are key components of the cartilage's extracellular matrix [49].

Another important biomarker of joint degeneration is VEGF. Elevated VEGF levels stimulate signaling pathways and promote angiogenesis, both processes that are reported to contribute to the onset and progression of OA [50, 51]. It has been reported that VEGF expression increases in the serum and within the joint during the progression of OA [38]. Lastly, EGF is a molecule critical for cell proliferation, tissue repair, and homeostasis, and the significant reduction of serum EGF in LCR rats fed the HFHS diet suggests a diminished ability of the LCR rats to respond to obesity‐induced inflammation [52]. The overall metabolic profile in the LCR rats, similar to what has been observed in SD rats, points to systemic metabolic dysfunction potentially contributing to the degenerative changes of the knee cartilage detected in the present study [16, 35, 48]. In contrast, the metabolic profile in the HCR rats, characterized by reduced levels of pro‐inflammatory cytokines, such as IL‐1β and VEGF, suggests a more protective environment associated with intrinsic high aerobic capacity, thereby potentially contributing to the maintenance of knee joint integrity in the HCR animals. Thus, similarities and differences between LCR, HCR, and SD rats in their responses to the HFHS diets have been observed. Some of these differences likely reflect the broader set of genetic and metabolic traits that distinguish these selectively bred strains of rats, in addition to the difference in intrinsic aerobic capacity.

After 12 weeks of the diet intervention, both HCR and LCR rats fed the HFHS diet had elevated blood glucose levels. However, during the OGTT test, the HCR rats, regardless of diet, returned to baseline blood glucose levels within 120 min, while the LCR rats, regardless of diet, remained hyperglycemic at the same time point. Chronic hyperglycemia is a hallmark of metabolic syndrome and potentially contributes to OA pathogenesis through oxidative stress and formation of advanced glycation end products that damage cartilage matrix and subchondral bone [11]. Therefore, impaired glucose tolerance in the LCR rats likely reflects reduced mitochondrial oxidative capacity, one of several metabolic traits that distinguish LCR from HCR rats, which may result in the downstream complications of hyperglycemia‐driven damage, contributing to loss of joint integrity [11].

This study is the first to demonstrate the association of intrinsic aerobic capacity with the maintenance of knee joint integrity, specifically focusing on the articular cartilage, synovial membrane, IFP, and subchondral bone. By linking intrinsic aerobic capacity to joint degeneration risk, in the future, one can potentially identify genetic biomarkers associated with intrinsic aerobic capacity as genetic biomarkers that can be targeted, supporting interventions via aerobic exercise training or pharmacological enhancement aimed at modulating aerobic capacity. Interestingly, the intrinsic differences between HCR and LCR rats may parallel variability seen in humans, where some individuals, often high‐performing athletes but not exclusive to them, naturally possess greater aerobic capacity and metabolic resilience than others [53]. Athletes tend to exhibit lower baseline inflammation and superior metabolic regulation, and individuals with lower intrinsic aerobic fitness are more susceptible to chronic inflammation, impaired glucose handling, and associated structural complications [53]. These findings suggest that intrinsic aerobic capacity in rats, much like in humans, may be a key determinant of protection against metabolic and inflammatory stressors.

When interpreting the results of the studies presented, certain limitations should be kept in mind. First, the studies were conducted only in male LCR and HCR rats. It remains to be determined if similar differences in obesity‐induced metabolic disturbance and knee joint integrity are also observed in female LCR and HCR group rats. Previous studies with male and female SD rats indicated that aspects of the response to an HFHS diet are sex‐specific [21]. Second, the rats were only fed the HFHS diet for 12 weeks. Future studies should explore longer diet intervention periods to assess the effects of an HFHS diet longitudinally and after more chronic exposure. Third, the animals were not exercised during the studies, and previous studies have indicated exercise in SD rats led to resistance to the adverse effects of the HFHS diet on joint integrity, and therefore, exercise may overcome the metabolic dysfunction in the LCR rats [18, 19].

In summary, the results of this study lead to the conclusion that the knee joints of N:NIH rats with a high intrinsic aerobic capacity are better protected against a dietary challenge that perturbs the metabolic profile than rats with a low intrinsic aerobic capacity. Aerobic capacity is a genetically determined factor that can be increased/improved by systematic aerobic exercise training. If improved aerobic capacity acquired via training is equally successful in protecting the system from problems associated with diets rich in fat and sugar as high intrinsic aerobic capacity remains to be studied.

## Author Contributions

Darsh Tripathi was responsible for the interpretation of data, drafting the manuscript, contributed to the execution of the study, revising the manuscript, and approving the final version. Mauricio Delgado‐Bravo contributed to the execution of the study, data collection, revising the manuscript, and approving the final submitted version. Ruth A. Seerattan and Venus Joumaa contributed to the design of the study, execution of the study, revising the manuscript, and approving the final submitted version. David A. Hart and Raylene A. Reimer contributed to the interpretation of data, revising the manuscript, and approving the final version. Lauren Gerard Koch contributed to the design of the study, resources, review and editing the manuscript, and approving the final version. Walter Herzog was responsible for the design of the study, execution of the study, data collection, data analysis, interpretation of data, revising the manuscript, and approving the final version.

## Conflicts of Interest

The authors declare no conflicts of interest.

## Supporting information


Supporting File

## Data Availability

The data that support the findings of this study are available from the corresponding author upon reasonable request.
