# Supplementary material for: Variation in Intrinsic Aerobic Capacity Reveals Distinct Differences in the Maintenance of Rat Knee Joint Integrity
Source: J Orthop Res. 2026 Oct 19;44(10):e70299. doi: 10.1002/jor.70299 (PMC13679882; doi:10.1002/jor.70299)
Supplement: Supplementary file 1 [file JOR-44-0-s001.docx]

**Supplementary Material**

Table 1: Subchondral bone parameters measured using a micro-CT system. Data are presented as means (CI). The strain p-values are calculated by collapsing across both diets, and the diet p-values are calculated by collapsing across both strains.

|  | LCR-Chow | LCR-HFHS | HCR-Chow | HCR-HFHS | Two-Way Anova | | | | | |
| --- | --- | --- | --- | --- | --- | --- | --- | --- | --- | --- |
|  |  |  |  |  | Strain (S) | | Diet (D) | | S*D | |
|  |  |  |  |  | F | p-value | F | p-value | F | p-value |
| Total Volume (mm^3^) | 149.6 (142.7-156.5) | 153.8 (144.1-163.5) | 129.3 (122.6-136.0) | 136.7 (126.9-146.6) | 24.11 | **<0.0001** | 2.3 | 0.14 | 0.18 | 0.67 |
| Bone Volume (mm^3^) | 109.2 (102.8-115.5) | 106.4 (98.3-114.5) | 85.7 (81.4-90.0) | 86.5 (82.5-90.6) | 64.16 | **<0.0001** | 0.13 | 0.72 | 0.42 | 0.52 |
| Bone Surface (mm^2^) | 628.9 (588.8-669.0) | 677.1 (630.1-724.2) | 694.5 (553.8-635.2) | 645.7 (580.4-711.12=) | 2.25 | 0.14 | 5.14 | **0.03** | 0.00 | 0.95 |
| Bone Volumetric Fraction (%) | 73.0 (70.5-75.5) | 69.1 (674-70.9) | 66.4 (63.9-68.9) | 63.6 (60.0-67.2) | 27.40 | **<0.001** | 8.10 | **0.008** | 0.21 | 0.65 |
| Bone Mineral Density (mg HA/cm^3^) | 644.4 (625.1-663.6) | 615.5 (598.6-632.5) | 585.2 (566.9-603.5) | 563.8 (537.3-590.3) | 38.27 | **<0.0001** | 7.84 | **0.009** | 0.17 | 0.68 |
| Specific Bone Surface (mm^-1^) | 5.8 (5.3-6.2) | 6.4 (6.0-6.7) | 6.9 (6.5-7.4) | 7.5 (6.8-8.1) | 28.61 | **<0.0001** | 7.15 | **0.01** | 0.06 | 0.82 |
| Trabecular Number (mm^-1^) | 3.4 (3.3-3.5) | 3.3 (3.2-3.  4) | 3.5 (3.3-3.6) | 3.4 (3.2-3.6) | 1.71 | 0.20 | 0.99 | 0.33 | 0.01 | 0.92 |
| Trabecular Thickness (mm) | 0.31 (0.29-0.32) | 0.29 (0.28-0.30) | 0.27 (0.26-0.28) | 0.26 (0.25-0.28) | 32.98 | **<0.0001** | 4.45 | **0.04** | 0.28 | 0.60 |
| Trabecular Separation (mm) | 0.24 (0.23-0.26) | 0.26 (0.24-0.27) | 0.27 (0.25-0.29) | 0.28 (0.26-0.30) | 9.01 | **0.005** | 2.43 | 0.13 | 0.12 | 0.73 |
| Connectivity Density (1/mm^3^) | 11.3 (9.6-13.0) | 11.9 (10.9-13.0) | 13.8 (12.0-15.7) | 14.3 (12.5-16.1) | 12.51 | **0.001** | 0.62 | 0.44 | 0.01 | 0.92 |
| Tissue Mineral Density (mg HA/cm^3^) | 901.2 (868.2-934.3) | 908.0 (876.6-939.3) | 881.6 (857.9-905.4) | 884.4 (856.5-912.3) | 2.82 | 0.10 | 0.14 | 0.72 | 0.02 | 0.88 |

Table 2: Serum 22 cytokine/adipokine profile for HCR and LCR rats fed a HFHS and Chow diet at the end of the experimental diet intervention. Data are presented as means ± 1 SEM. The strain p-values are calculated by collapsing across both diets, and the diet p-values are calculated by collapsing across both strains.

|  | LCR-Chow | LCR-HFHS | HCR-Chow | HCR-HFHS | Two-Way Anova | | | | | |
| --- | --- | --- | --- | --- | --- | --- | --- | --- | --- | --- |
|  |  |  |  |  | Strain (S) | | Diet (D) | | S*D | |
|  |  |  |  |  | F | p-value | F | p-value | F | p-value |
| Eotaxin | 21.7 ± 3.4 | 15.8 ± 2.3 | 20.5 ± 1.7 | 12.5 ± 2.6 | 0.69 | 0.41 | 6.6 | **0.02** | 0.17 | 0.67 |
| GM-CSF | 184.3 ± 31.2 | 155.3 ± 16.8 | 173.4 ± 17.9 | 90.7 ± 16.2 | 3.18 | **0.09** | 6.96 | **0.01** | 1.61 | 0.22 |
| EGF | 347.9 ± 122.8 | 50.2 ± 15.8 | 107.5 ± 64.9 | 155.7 ± 67.5 | 0.88 | 0.36 | 3.01 | 0.1 | 5.79 | **0.024** |
| Fractalkine | 151.2 ± 11.3 | 122.1 ± 9.7 | 134.4 ± 9.5 | 117.3 ± 11.6 | 0.97 | 0.33 | 4.44 | **0.04** | 0.31 | 0.59 |
| G-CSF | 156.1 ± 33.3 | 105.1 ± 15.2 | 147.3 ± 11.9 | 99.2 ± 19.1 | 0.12 | 0.74 | 5.4 | **0.03** | <0.01 | 0.95 |
| IFNγ | 498.5 ± 113.1 | 498.9 ± 50.3 | 593.1 ± 57.9 | 340.3 ± 93.2 | 1.86 | 0.18 | 6.53 | **0.02** | 0.41 | 0.53 |
| IL-1β | 278.4 ± 41.2 | 332.3 ± 64.4 | 246.5 ± 27.3 | 149.1 ± 32.9 | 5.94 | **0.02** | 2.74 | 0.11 | 2.03 | 0.17 |
| IL-2 | 436.8 ± 89.8 | 401.9 ± 42.2 | 392.5 ± 53.2 | 229.4 ± 70.4 | 2.76 | 0.11 | 2.3 | 0.14 | 0.96 | 0.34 |
| IL-4 | 182.9 ± 31.1 | 164.0 ± 18.6 | 160.8 ± 8.0 | 106.4 ± 24.8 | 2.96 | 0.1 | 2.51 | 0.13 | 0.59 | 0.45 |
| IL-5 | 169.5 ± 12.7 | 181.9 ± 12.8 | 163.8 ± 15.0 | 127.0 ± 26.3 | 2.77 | 0.11 | 0.45 | 0.51 | 1.83 | 0.19 |
| IL-6 | 2491.2 ± 617.8 | 2144.7 ± 328.8 | 1488.1 ± 487.7 | 1104.5 ± 282.2 | 4.08 | 0.05 | 5.76 | **0.02** | 0.09 | 0.77 |
| IL-12p70 | 614.3 ± 91.8 | 476.9 ± 68.7 | 537.3 ± 64.5 | 399.0 ± 95.4 | 2.41 | 0.13 | 1.13 | 0.3 | 0.38 | 0.54 |
| IL-13 | 105.1 ± 23.5 | 61.4 ± 11.9 | 76.5± 8.9 | 39.3 ± 7.7 | 3.14 | 0.09 | 7.98 | **0.01** | 0.05 | 0.82 |
| IL-17A | 145.7 ± 20.7 | 150.8 ± 13.7 | 132.3 ± 33.6 | 117.1 ± 31.4 | 0.9 | 0.35 | 0.04 | 0.84 | 0.17 | 0.69 |
| IP-10 | 414.4 ± 32.9 | 324.9 ± 27.5 | 239.4 ± 25.6 | 410.1 ± 57.8 | 1.27 | 0.27 | 1.04 | 0.32 | 10.68 | **<0.01** |
| LIX | 5.2 ± 0.4 | 3.3 ± 0.2 | 1.8 ± 0.2 | 3.0 ± 0.3 | 34.77 | **<0.01** | 1.28 | 0.27 | 24.43 | **<0.01** |
| MCP-1 | 1316.0 ± 188.2 | 1388.2 ± 111.6 | 1312.9 ± 112.4 | 908.8 ± 182.7 | 2.41 | 0.13 | 1.15 | 0.29 | 2.36 | 0.14 |
| MIP-1α | 28.3 ± 2.3 | 24.5 ± 1.5 | 25.7± 1.9 | 23.8 ± 2.2 | 0.72 | 0.41 | 2 | 0.17 | 0.21 | 0.65 |
| MIP-2 | 56.8 ± 11.5 | 44.4 ± 9.7 | 47.4 ± 6.1 | 29.3 ± 3.8 | 1.86 | 0.19 | 2.86 | 0.1 | 0.11 | 0.75 |
| RANTES | 2.4 ± 0.3 | 82.2 ± 80.3 | 391.8 ± 176.0 | 183.1 ± 126.1 | 1.22 | 0.28 | 0 | 0.97 | 2.68 | 0.11 |
| TNFα | 19.1 ± 5.1 | 18.2 ± 2.6 | 19.2± 1.3 | 13.6± 3.1 | 0.43 | 0.52 | 0.9 | 0.35 | 0.49 | 0.49 |
| VEGF | 184.4 ± 11.2 | 118.8 ± 15.3 | 125.8 ± 11.2 | 113.2 ± 13.8 | 5.11 | **0.03** | 7.59 | **0.01** | 3.48 | 0.07 |
